# Transcatheter Therapy in Secondary Mitral Regurgitation: Recent Clinical Evidence and Emerging Frontiers

**DOI:** 10.7759/cureus.106989

**Published:** 2026-04-13

**Authors:** Álvaro Torres, Renato Jochava, Francisca Quintanilla, Catalina Córdova, Günther Krögh, Santiago Besa

**Affiliations:** 1 School of Medicine, Faculty of Medicine, Pontificia Universidad Católica de Chile, Santiago, CHL; 2 Department of Cardiac Surgery, Division of Surgery, Faculty of Medicine, Pontificia Universidad Católica de Chile, Santiago, CHL

**Keywords:** chronic heart failure, functional mitral regurgitation, heart team, mitraclip®, mitral valve insufficiency, secondary mitral regurgitation (smr), structural heart disease intervention, transcatheter edge-to-edge repair, transcatheter mitral valve replacement, valvular-heart disease

## Abstract

Secondary mitral regurgitation (SMR) is a common valvular disorder in patients with heart failure and is associated with poor prognosis. Transcatheter edge-to-edge repair (TEER), particularly with the MitraClip device, has emerged as a less invasive alternative to surgery, with growing evidence supporting its clinical role. However, trial results have been heterogeneous, and optimal patient selection remains under debate.

A narrative literature review was conducted using a targeted manual search of PubMed (2018-2026), with the terms (“secondary mitral regurgitation” OR “functional mitral regurgitation”) AND (transcatheter OR MitraClip) AND (randomized OR trial OR meta-analysis). A total of 13 key studies were included, comprising randomized clinical trials, meta-analyses, and relevant prospective series, complemented by 30 selected supporting studies used for contextualization.

Recent trials such as COAPT (Cardiovascular Outcomes Assessment of the MitraClip Percutaneous Therapy for Heart Failure Patients With Functional Mitral Regurgitation) and RESHAPE-HF2 (Randomized Study of the MitraClip Device in Heart Failure Patients with Clinically Significant Functional Mitral Regurgitation) suggest that TEER reduces heart failure hospitalizations in selected patients, while MATTERHORN (Multicenter Mitral Valve Reconstruction for Advanced Insufficiency of Functional or Ischemic Origin) supports transcatheter repair as a safer short-term alternative to surgery in appropriate cases. The concept of disproportionate mitral regurgitation has become central to identifying candidates most likely to benefit. Alternative devices such as PASCAL (Edwards Lifesciences, Irvine, California) and emerging technologies, including transcatheter mitral valve replacement (TMVR), are expanding therapeutic options.

TEER is a valuable and safe therapeutic option for patients with SMR who remain symptomatic despite optimal medical therapy, particularly when careful clinical and anatomical selection is applied. Although this review has methodological limitations inherent to its narrative design, it provides an updated and clinically relevant overview of current evidence and future directions in SMR management.

## Introduction and background

Secondary mitral regurgitation (SMR), also referred to as functional mitral regurgitation (MR), is a common complication in patients with left ventricular (LV) dysfunction [1]. It is observed in approximately one-third of patients with chronic heart failure (HF) and contributes to disease progression and worse prognosis [2]. Moderate to severe MR in HF is associated with a higher risk of recurrent hospitalizations and an annual mortality of around 5% or higher, depending on severity [2]. SMR results from ventricular (or left atrial) dilation and dysfunction, which alter valvular geometry and impair coaptation of structurally normal mitral leaflets [3].

Historically, SMR management has been based on optimization of guideline-directed medical therapy for HF, including pharmacological treatment and cardiac resynchronization when indicated, with surgical correction considered in selected cases [4]. However, unlike primary MR, mitral valve repair or replacement in SMR has not been shown to improve survival, particularly in patients with severe systolic dysfunction [2]. In addition, many patients are considered at high surgical risk due to advanced ventricular impairment and comorbidities, and prior to transcatheter therapies, severe SMR in nonsurgical patients often remained untreated, contributing to ongoing HF progression and poor prognosis [3,5,6].

Over the past decade, transcatheter valve therapies have expanded the treatment options for valvular disease [3]. In the mitral field, transcatheter edge-to-edge repair (TEER) with the MitraClip device has emerged as an option for patients considered inoperable or at prohibitive surgical risk [3,6]. After demonstrating safety and efficacy in degenerative MR, its use expanded to functional MR [3]. Early randomized experience came from the EVEREST II (Endovascular Valve Edge-to-Edge Repair Study II) trial, which showed a more favorable early safety profile than surgery but less complete reduction of MR, although it included predominantly patients with degenerative rather than SMR [7]. In 2018, two pivotal randomized controlled trials evaluating MitraClip in SMR reported discordant findings: MITRA-FR (Percutaneous Repair With the MitraClip Device for Severe Secondary Mitral Regurgitation) showed no clinical benefit from adding MitraClip to medical therapy [5], whereas COAPT (Cardiovascular Outcomes Assessment of the MitraClip Percutaneous Therapy for Heart Failure Patients With Functional Mitral Regurgitation) reported significant reductions in HF hospitalizations and mortality [6]. These discrepancies intensified the debate regarding which patients with SMR derive the greatest benefit from percutaneous intervention [8].

Subsequent RCTs and meta-analyses have further clarified the role of transcatheter therapy in SMR, including RESHAPE-HF2 (Randomized Study of the MitraClip Device in Heart Failure Patients with Clinically Significant Functional Mitral Regurgitation), designed as a "tie-breaker" between COAPT and MITRA-FR [9,10], and MATTERHORN (Multicenter Mitral Valve Reconstruction for Advanced Insufficiency of Functional or Ischemic Origin), which directly compared transcatheter repair with surgery [11]. In parallel, the TEER landscape has expanded beyond MitraClip to include other systems such as PASCAL (Edwards Lifesciences, Irvine, California), while transcatheter mitral valve replacement (TMVR) remains under active clinical investigation [10,12,13]. Given this rapidly evolving evidence, an updated review of transcatheter therapies for SMR is warranted.

Therefore, the objective of this narrative review is to synthesize recent evidence on transcatheter therapies for SMR, focusing on major RCTs, meta-analyses, TEER platforms such as MitraClip and PASCAL, and the emerging role of TMVR.

Literature search strategy

A targeted narrative review was conducted to synthesize contemporary evidence on transcatheter therapies for SMR. PubMed was used as the primary database, and the last search was performed on March 16, 2026. The primary search strategy was: ("secondary mitral regurgitation" OR "functional mitral regurgitation") AND (transcatheter OR MitraClip) AND (randomized OR trial OR meta-analysis). The main search focused on studies published between 2018 and 2026. Eligible core studies included randomized controlled trials, key follow-up analyses, contemporary meta-analyses, and selected clinically relevant prospective studies in adults.

The 13 core studies were identified through the primary PubMed search and selected qualitatively based on whether they addressed one or more of the main clinical domains covered in this review, including mortality, HF hospitalization, procedural safety, comparative effectiveness, echocardiographic response, reverse remodeling, and patient selection. Google Scholar was used only as a supplementary source to identify supporting references and related articles. Selected international society guidelines cited in the bibliography were included only for contextualization of current recommendations. These supplementary sources did not contribute additional core studies beyond those identified through the primary PubMed search.

Studies focused predominantly on primary or degenerative MR, case reports, editorials, narrative commentaries, and conference abstracts without sufficient full-text data were not included as core evidence. However, a limited number of non-SMR platform-specific studies were retained when considered useful for contextualizing device-related discussion.

**Figure 1:**
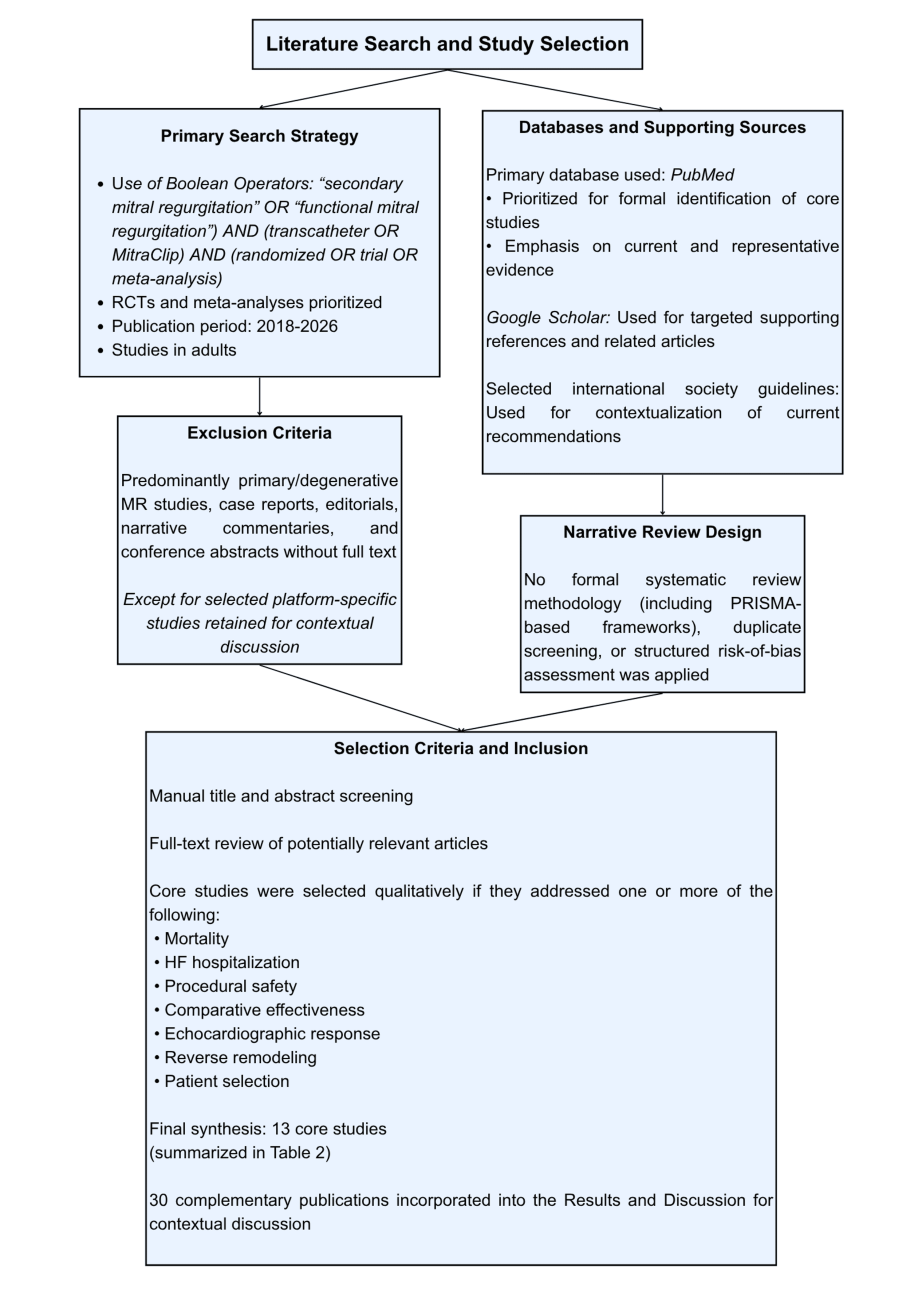
Search Strategy and Study Selection Process Overview of the literature search approach, data sources, and study selection process used in this narrative review. RCT, randomized controlled trial; TEER, transcatheter edge-to-edge repair; SMR, secondary mitral regurgitation.

Titles and abstracts were screened manually, followed by full-text review of potentially relevant articles. The final synthesis included 13 core studies and 30 complementary publications. As this was a narrative review, no formal systematic review methodology, including PRISMA-based or related frameworks, was applied. Duplicate screening and structured risk-of-bias assessment were not performed. These limitations were assumed from the outset and are inherent to the narrative design of the study. The study selection process is summarized in Figure 1.

## Review

Included studies

This review included four major RCTs (MITRA-FR, COAPT, RESHAPE-HF2, and MATTERHORN), together with key follow-up analyses, contemporary meta-analyses, and selected studies on alternative TEER platforms and TMVR. In total, 13 core studies were included. In addition, 30 complementary publications were incorporated into the Results and Discussion sections to expand interpretation in areas such as patient-centered outcomes, reverse remodeling, subgroup analyses, and clinical decision-making. The main thematic areas addressed in this review are summarized in Table 1.

**Table 1:** Main Thematic Areas Addressed in This Review This table summarizes the principal thematic areas addressed in this review, the predominant type of evidence supporting each area, and their specific contribution to the overall discussion of transcatheter therapy in secondary mitral regurgitation. TEER, transcatheter edge-to-edge repair; SMR, secondary mitral regurgitation; HF, heart failure; MR, mitral regurgitation; TMVR, transcatheter mitral valve replacement.

Main Thematic Area	Predominant Evidence Type	Role in the Review
TEER versus medical therapy in SMR	Randomized trials and pooled analyses	Provides the main comparative framework for efficacy, especially regarding HF hospitalization and mortality.
TEER versus surgery	Randomized and pooled comparative evidence	Supports discussion of relative safety, completeness of MR reduction, and individualized treatment selection.
Patient selection and clinical modifiers	Secondary analyses, subgroup studies, and supporting reviews	Explores which anatomical and clinical factors may influence response to TEER.
Alternative transcatheter platforms and TMVR	Comparative, prospective, and early investigational evidence	Provides context on platform expansion and emerging treatment options beyond MitraClip.
Long-term outcomes and decision-making perspective	Follow-up studies, meta-analytic evidence, and guideline-based context	Complements the review with durability, reverse remodeling, and practical clinical integration.

Transcatheter repair versus medical therapy

Pivotal Randomized Trials

The pivotal randomized trials evaluating TEER in SMR have shown contrasting results, with their main characteristics and principal findings summarized in Table 2. COAPT demonstrated a clear benefit of MitraClip added to optimal medical therapy, with significant reductions in HF hospitalizations and all-cause mortality in selected patients with severe, symptomatic SMR [6]. In contrast, MITRA-FR did not show a clinical benefit of adding MitraClip to medical therapy in a population with severe SMR [5]. More recently, RESHAPE-HF2 supported a benefit of transcatheter repair over medical therapy, driven mainly by a reduction in HF-related events, although without a statistically significant reduction in isolated mortality at the reported follow-up [10]. Taken together, these trials suggest that the most consistent and reproducible benefit of TEER over medical therapy is a reduction in HF hospitalization, whereas mortality effects remain less uniform across studies [5,6,10].

Contemporary Pooled Evidence

Contemporary pooled analyses have generally reinforced the signal observed in the randomized trials. Meta-analyses incorporating COAPT, MITRA-FR, and RESHAPE-HF2 consistently showed that TEER reduces HF hospitalization and improves composite clinical outcomes compared with medical therapy alone, while not demonstrating a clear and statistically consistent reduction in all-cause or cardiovascular mortality in the main analyses [9,14]. Additional pooled data, including reconstructed time-to-event analyses and broader meta-analytic cohorts, have also suggested improvement in functional status and possible long-term survival benefit, although these findings are less uniform and often depend on study design, follow-up duration, and inclusion of nonrandomized data [15,16]. Overall, pooled evidence supports reduction in HF-related clinical burden as the most robust effect of TEER in SMR, whereas any survival advantage remains more variable and should be interpreted with caution [9,14-16].

Patient-Centered and Complementary Outcomes

Beyond traditional hard endpoints, several analyses have provided complementary evidence supporting the clinical value of TEER. A patient-centered analysis derived from COAPT showed that, although days at home were similar between groups at one year, patients treated with TEER spent more days at home at two years than those receiving medical therapy alone [17]. Earlier COAPT analyses also showed improved health status after TEER [18,19]. In addition, a dedicated analysis from RESHAPE-HF2 demonstrated that TEER reduced recurrent HF hospitalizations, both when analyzed alone and when combined with cardiovascular death, and was also associated with improved quality of life [20]. New York Heart Association (NYHA) class improvement was also consistent in COAPT [21]. Consistent with this, a COAPT analysis showed that TEER reduced both fatal and nonfatal HF hospitalizations and increased time alive out of hospital compared with GDMT alone, further emphasizing the prognostic importance of reducing recurrent congestion and hospital admission burden [22].

Reverse Remodeling and Echocardiographic Response

Structural and echocardiographic data also support a beneficial effect of TEER in SMR. A 2026 systematic review and meta-analysis of 42 studies including 3,987 patients found that TEER was associated with significant reverse remodeling, including reductions in LV end-diastolic and end-systolic volumes and diameters, improved right ventricular function, lower pulmonary artery systolic pressure, and a modest increase in LV ejection fraction. No significant improvement was observed in global longitudinal strain or left atrial end-systolic volume [23]. Consistent with this, a COAPT substudy showed that LV reverse remodeling at six months was associated with improved subsequent two-year outcomes, although the extent of remodeling was not clearly modified by TEER itself or by the degree of residual MR [24]. In addition, echocardiographic follow-up from COAPT demonstrated more favorable structural and hemodynamic changes after TEER, including greater MR reduction and evidence of reverse remodeling, compared with guideline-directed medical therapy alone [25].

Transcatheter repair versus conventional surgery

Historically, many patients with SMR were not referred for isolated mitral valve surgery because of the lack of clear evidence for benefit in hard outcomes. MATTERHORN, the first randomized trial directly comparing transcatheter repair with surgery in this setting, showed that MitraClip was non-inferior to surgery for the composite efficacy endpoint at one year, while also demonstrating a substantially more favorable early safety profile [11]. These findings suggest that, in selected surgical candidates with SMR, TEER may achieve comparable short-term clinical outcomes with lower early procedural risk. This safety signal is also consistent with prior TEER experience, including COAPT, in which most patients were free of device-related complications at one year [6,11].

Alternative TEER platforms 

Beyond MitraClip, additional TEER platforms such as PASCAL have expanded the transcatheter treatment landscape. In high-risk patients with primary MR, the CLASP IID trial showed non-inferiority of PASCAL compared with MitraClip, with no significant differences in survival, HF rehospitalization, or major adverse events at one year [26]. Similarly, a meta-analysis of mainly observational comparative studies found no significant differences between PASCAL and MitraClip in procedural success, complication rates, or achieved MR reduction [12]. Although CLASP IID focused on primary MR, these data support PASCAL as a relevant alternative TEER platform.

Overall clinical perspective

Overall, the available evidence supports transcatheter therapy, particularly MitraClip, as an established treatment option for carefully selected patients with SMR rather than an experimental intervention. Across randomized and pooled evidence, the most consistent clinical benefit has been reduction in HF hospitalization [6,9]. By contrast, survival benefits have been less uniform: COAPT provided the strongest signal for mortality reduction [6], whereas MITRA-FR did not show a mortality benefit and pooled analyses have reported only a nonsignificant overall trend [5,9]. Taken together, these findings suggest that the clinical impact of TEER in SMR is not homogeneous and depends largely on appropriate patient selection and on the underlying ventricular and pathophysiological profile of each case.

Patient selection: proportionate versus disproportionate regurgitation

Recent publications have emphasized the clinical value of detailed anatomical and functional characterization in patients with SMR, particularly the importance of interpreting MR severity in relation to ventricular size and remodeling when selecting candidates for transcatheter treatment [8,27]. In this context, the conceptual framework of “proportionate vs. disproportionate regurgitation” proposed by Grayburn et al. has been particularly useful in explaining the heterogeneous results of TEER in SMR [8].

According to this framework, MR may be proportionate to the degree of LV dilation, with a large regurgitant orifice largely explained by advanced ventricular remodeling, or disproportionate, with severe MR despite only moderately increased LV size [8]. This distinction has been used to interpret the discrepant findings of MITRA-FR and COAPT, suggesting that the former predominantly included patients with proportionate MR, whereas the latter selected patients with disproportionately severe MR, in whom valve correction may have greater clinical impact [8]. Supporting this interpretation, a landmark MITRA-FR analysis suggested delayed benefit on HF hospitalization [28], and a subsequent editorial analyzing the three major trials highlighted LV end-diastolic volume as a potential modifier of the mortality effect of repair [10].

Further analyses have reinforced the value of integrating MR severity, ventricular size, and overall clinical profile in patient selection. A secondary analysis of COAPT showed that the relationship between effective regurgitant orifice area and LV end-diastolic volume was associated with clinical outcomes after TEER [29]. In addition, severe LV dysfunction was associated with worse post-TEER outcomes [30], while another COAPT analysis showed that baseline clinical profile and early post-procedural changes in MR severity and right ventricular systolic pressure were associated with long-term clinical response [27]. Taken together, these findings support the concept that patients with disproportionately severe MR are more likely to derive meaningful benefit from TEER, particularly when valve intervention can interrupt the cycle of volume overload, adverse remodeling, and recurrent HF.

Combination with optimal medical therapy

All trials indicated optimization of guideline-directed medical therapy (GDMT) before and after intervention. However, it is worth noting that these RCTs were designed in the prior decade, before widespread adoption of newer drug classes such as SGLT2 inhibitors. Prognosis in HF may therefore have improved since then, potentially reducing the incremental benefit of MitraClip in the context of more potent therapies. None of the trials systematically integrated the current “four pillars” of HF treatment (ACEI/ARB/ARNI, beta-blockers, mineralocorticoid antagonists, and SGLT2 inhibitors) across all patients [10]. This leaves an open question: under contemporary pharmacologic therapy, could some patients previously eligible for MitraClip stabilize without invasive intervention? Still, in real-world clinical practice, transcatheter therapy is considered complementary rather than competitive to medical therapy. The strategy remains: maximize ventricular optimization with GDMT, and if ≥3+ MR with persistent symptoms remains, then MitraClip is a reasonable next step. For example, in RESHAPE-HF2, all patients were on maximally tolerated medications, yet intervention still improved outcomes [10].

Key benefits

Reduction in HF hospitalization remains the most consistent benefit of MitraClip across studies. This overall direction is also supported by earlier pooled evidence, as a 2023 systematic review and meta-analysis suggested more favorable long-term morbidity and mortality outcomes with percutaneous mitral valve repair than with medical therapy or surgery, although the included data were heterogeneous and derived largely from nonrandomized studies [31]. Beyond hospitalization, TEER has also been associated with improvement in functional capacity and quality of life [8], and a COAPT analysis showed that early improvement in health status after TEER was associated with better subsequent clinical outcomes [19].

By contrast, mortality benefits have been less consistent. COAPT provided a clear signal of survival benefit [8], whereas RESHAPE-HF2 did not detect a mortality difference at the reported follow-up [10]. Still, the five-year follow-up of COAPT supported the durability of benefit, with lower all-cause mortality and a lower combined risk of death or HF hospitalization in the MitraClip group [32]. Overall, the available evidence suggests that TEER most reproducibly reduces HF-related clinical burden, while any survival advantage appears more dependent on follow-up duration and patient selection [8,10,32].

Patient selection for surgery

TEER Versus Surgery in Operable Patients

The comparison between transcatheter repair and surgery has important implications for patient selection, and earlier network meta-analytic evidence also provides useful context for this therapeutic question [33]. Although MATTERHORN suggested short-term clinical equivalence between TEER and surgery, these findings should be interpreted with caution, given the limited follow-up currently available [11]. In the updated systematic review and meta-analysis by Pinilla et al., MitraClip was less likely to achieve MR ≤2+ both immediately after the procedure and at follow-up, whereas no significant differences were observed in all-cause mortality, cardiovascular mortality, HF hospitalizations, or NYHA functional status between transcatheter and surgical strategies [34].

Taken together, these findings support an individualized approach rather than a uniform preference for one strategy. Surgery may remain preferable when anatomy favors a more complete or durable correction or when concomitant surgical treatment is required, whereas TEER may be particularly attractive when a less invasive approach is preferred or when the broader clinical scenario limits surgical candidacy [11,34].

Repeat mitral intervention after TEER should also be considered when discussing durability [35]. In addition, the heterogeneity of surgical management in pooled studies and the fragility of some sensitivity analyses indicate that the current comparative evidence remains limited, reinforcing the need for careful patient selection and longer-term follow-up [34]. Recent reviews likewise support an individualized role for surgery when more complete or durable correction is expected [36].

Additional Clinical Modifiers of Candidate Selection

Several additional clinical factors may help refine candidate selection for TEER. Age alone should not preclude treatment, as a COAPT analysis showed reductions in death or HF hospitalization and improvements in survival and quality of life in both younger and older patients, although the reduction in HF hospitalization appeared less pronounced in older individuals [37].

Baseline HF burden and clinical profile may also influence risk stratification. A recent COAPT subgroup analysis showed that elevated natriuretic peptide levels and prior HF hospitalization identified patients at higher two-year risk, while the relative benefit of TEER on mortality and HF hospitalization remained consistent across different degrees of HF severity [38]. Etiology may also matter, particularly ischemic versus nonischemic SMR [39]. In the same direction, the COAPT risk score incorporates both clinical and echocardiographic variables and supports structured risk stratification during candidate selection [40].

Comorbidities and hemodynamic status may provide further refinement. Baseline renal dysfunction identified patients at higher two-year risk, although MitraClip improved outcomes regardless of renal function and reduced new-onset end-stage renal disease and the need for renal replacement therapy [41]. Baseline pulmonary hypertension may also assist risk stratification, although the benefit of TEER appeared preserved across hemodynamic subgroups [42]. Taken together, these findings support a more individualized approach to candidate selection that integrates anatomy, HF burden, comorbidity profile, and hemodynamic status rather than relying on a single variable alone.

Atrial functional MR as complementary evidence

In the related setting of atrial functional MR, a 2023 meta-analysis of observational studies showed that TEER achieved high rates of MR reduction and clinical outcomes comparable to those reported in ventricular functional MR, supporting the feasibility of transcatheter treatment in this subgroup, although prospective comparative data remain limited [43].

Although not directly representative of ventricular SMR, a recent meta-analysis in atrial functional MR reported similar short-term outcomes between surgery and TEER, whereas surgery appeared more favorable at longer follow-up for recurrent severe MR, all-cause mortality, HF hospitalization, and persistent NYHA III/IV symptoms. Given the heterogeneity of the included studies, these findings should be regarded as complementary and hypothesis-generating rather than definitive for SMR [44].

Transcatheter mitral valve replacement (TMVR)

TMVR represents a distinct transcatheter strategy and remains an investigational but rapidly evolving field. Ongoing studies continue to refine its safety, durability, anatomical feasibility, and comparative role relative to TEER and surgery. As experience grows and device development advances, TMVR may become an important complementary strategy, particularly for patients with anatomies less suitable for edge-to-edge repair or in whom clip-based repair is unlikely to provide durable benefit.

Early TMVR data, despite concerning mortality, should not be dismissed. These devices were implanted in extremely ill patients who were unlikely to survive without intervention. Achieving trivial MR in 85-90% of cases, together with marked functional improvement, supports the technical feasibility of percutaneous valve replacement [13]. With improved patient selection, such as avoiding small ventricles to reduce the risk of LV outflow tract obstruction and optimizing anticoagulation to minimize thrombosis, complications may decrease. Moreover, new transseptal devices are under development and may further improve procedural safety. In the medium term, TMVR could become an alternative for patients in whom edge-to-edge repair is insufficient. At present, however, TMVR remains investigational and is limited to selected cases within research protocols. A propensity score-matched comparison also suggested favorable TMVR outcomes versus medical therapy in selected patients, although this evidence remains observational [45]. The 13 key studies included in this review are summarized in Table 2 and provide the core evidence supporting this review.

**Table 2:** Summary of Key Studies on Transcatheter Therapies Relevant to Secondary Mitral Regurgitation Main randomized trials, meta-analyses, and selected prospective studies evaluating transcatheter treatment strategies in secondary mitral regurgitation, together with selected contextual studies on alternative TEER platforms and transcatheter mitral valve replacement. TEER, transcatheter edge-to-edge repair; TMVR, transcatheter mitral valve replacement; SMR, secondary mitral regurgitation; MR, mitral regurgitation; HF, heart failure; FMR, functional mitral regurgitation; DMR, degenerative mitral regurgitation.

Author	Year	Type	Population Origin	n	Intervention	Key Findings
Stone et al. [6]	2018	RCT (COAPT)	USA and Canada	614	MitraClip + Medical Therapy vs. Medical Therapy alone	MitraClip significantly reduced HF hospitalizations at 24 months (35.8% vs. 67.9% per year) and mortality (29% vs. 46% at two years) compared to medical therapy alone.
Obadia et al. [5]	2018	RCT (MITRA-FR)	France	304	MitraClip + Medical Therapy vs. Medical Therapy alone	No differences in mortality or HF hospitalizations at 12 months between MitraClip + medical therapy vs. medical therapy alone in severe SMR (negative trial).
Anker et al. [10]	2024	RCT (RESHAPE-HF2)	Multinational (Europe and USA)	505	MitraClip + Medical Therapy vs. Medical Therapy alone	MitraClip + medical therapy reduced HF events (first and recurrent hospitalizations) and improved the combined endpoint of cardiovascular death or hospitalization vs. medical therapy. No significant reduction in isolated mortality at ~2 years.
Baldus et al. [11]	2024	RCT (MATTERHORN)	Germany	210	MitraClip vs. Surgery	Transcatheter repair was non-inferior to surgery at one year in patients with SMR (similar rates of death, rehospitalization, HF, stroke, etc.). MitraClip had a lower rate of major 30-day complications (14.9% vs. 54.8%), indicating a superior safety profile.
Mata et al. [9]	2025	Meta-analysis of 3 RCTs	Multinational (USA, France, and Germany)	1426	TEER + Medical Therapy vs. Medical Therapy alone	Percutaneous mitral repair in SMR reduced the risk of HF hospitalization by ~35–40% vs. medical management. No statistically significant reduction in two-year mortality was observed in the pooled analysis of the 3 RCTs (favorable trend, HR -0.76, p=0.07).
Zahr et al. [26]	2023	RCT (CLASP IID)	International	300	PASCAL vs. MitraClip (primary MR)	The PASCAL device was non-inferior to MitraClip in high-risk patients with degenerative MR. At one year, there were no differences in survival, HF rehospitalization, or major adverse events between PASCAL and MitraClip (comparable efficacy and safety).
Hosseini et al. [12]	2023	Systematic review and meta-analysis (6 studies)	Mixed	—	PASCAL vs. MitraClip	PASCAL and MitraClip show similar efficacy and safety in transcatheter mitral repair. Both devices achieve high success rates (reduction of MR to ≤2+) with low complication rates and no significant differences in early clinical outcomes.
Müller et al. [13]	2021	Prospective cohort study	Germany	100	Tendyne TMVR	The Tendyne transcatheter valve eliminated MR (93% without ≥2+ MR at two years) and improved symptoms (89% in NYHA I-II at one year) in inoperable patients with severe MR. However, it showed high mortality: 9% at 30 days, 29% at one year, and 39% at two years (mainly within the first three months post-implantation).
Sasmita et al. [16]	2024	Meta-analysis (10 studies)	Multinational (USA, Europe, and Asia)	2533	MitraClip/TEER vs. Medical Therapy or No Mitral Intervention	MitraClip was associated with better long-term survival (RR 0.87, 95% CI 0.78–0.98), improved LV remodeling (mean reduction in LVEDV = -10.36 mL), though it did not significantly change LVEF or mortality when compared with surgical MV repair or replacement.
Pinilla et al. [34]	2025	Systematic review + updated meta-analysis (11 studies)	Multinational (North America and Europe)	1605	MitraClip vs. Surgery	In patients with SMR, MitraClip was associated with a lower likelihood of achieving MR ≤2+ at follow-up compared with surgery. However, no significant differences were observed in all-cause mortality, cardiovascular mortality, HF hospitalizations, or NYHA class I/II at follow-up. ICU length of stay was shorter with MitraClip.
Ammirabile et al. [14]	2026	Meta-analysis of 3 RCTs	Multinational (Europe and USA)	1422	TEER + GDMT vs. GDMT alone	In symptomatic moderate-to-severe functional MR, TEER + GDMT reduced the composite of death or first HF hospitalization at 24 months and also reduced HF hospitalization compared with GDMT alone. No significant differences were observed in all-cause or cardiovascular mortality at 24 months in the main analysis.
Lombardi et al. [23]	2026	Systematic review and meta-analysis (42 studies)	Multinational	3987	TEER for SMR	In SMR, TEER was associated with significant reverse remodeling, including reductions in LV end-diastolic and end-systolic volumes and diameters, improved right ventricular function, and lower pulmonary artery systolic pressure. A small increase in LVEF was observed, while no significant improvement was found in global longitudinal strain or left atrial end-systolic volume.
Stone et al. [32]	2023	five-year follow-up of RCT (COAPT)	USA and Canada	614	MitraClip + Medical Therapy vs. Medical Therapy alone	At five-year follow-up, transcatheter edge-to-edge repair remained associated with lower rates of HF hospitalization and reduced mortality compared with medical therapy alone, supporting the long-term durability of the COAPT trial results.

Current international guidelines

Current international guidelines recommend TEER in carefully selected patients with symptomatic severe SMR despite optimized guideline-directed medical therapy, particularly when anatomy is suitable and surgical risk is not favorable. In both American and European guidelines, TEER has gained a class IIa recommendation in appropriately selected cases, reflecting its growing role in contemporary SMR management [46,47].

Beyond formal recommendations, guideline implementation remains influenced by local expertise, access to structural heart programs, multidisciplinary Heart Team evaluation, and device availability. These factors are especially relevant in settings where transcatheter therapies may be limited by cost or infrastructure despite supportive evidence.

Proposed decision-making framework in SMR

Rather than proposing a prescriptive management algorithm, the evidence reviewed in this manuscript supports a practical framework for therapeutic decision-making in SMR. In general, optimization of guideline-directed medical therapy remains the initial step, followed by reassessment of symptoms, MR severity, ventricular remodeling, and candidacy for device- or surgery-based intervention. In selected patients who remain symptomatic despite cardiac resynchronization therapy, TEER may still be considered, as a 2024 systematic review and meta-analysis in CRT non-responders reported meaningful MR reduction and functional improvement after intervention [48]. This is consistent with a COAPT analysis showing that the prognostic benefit of MitraClip over GDMT was preserved regardless of prior CRT implantation [49].

**Figure 2:**
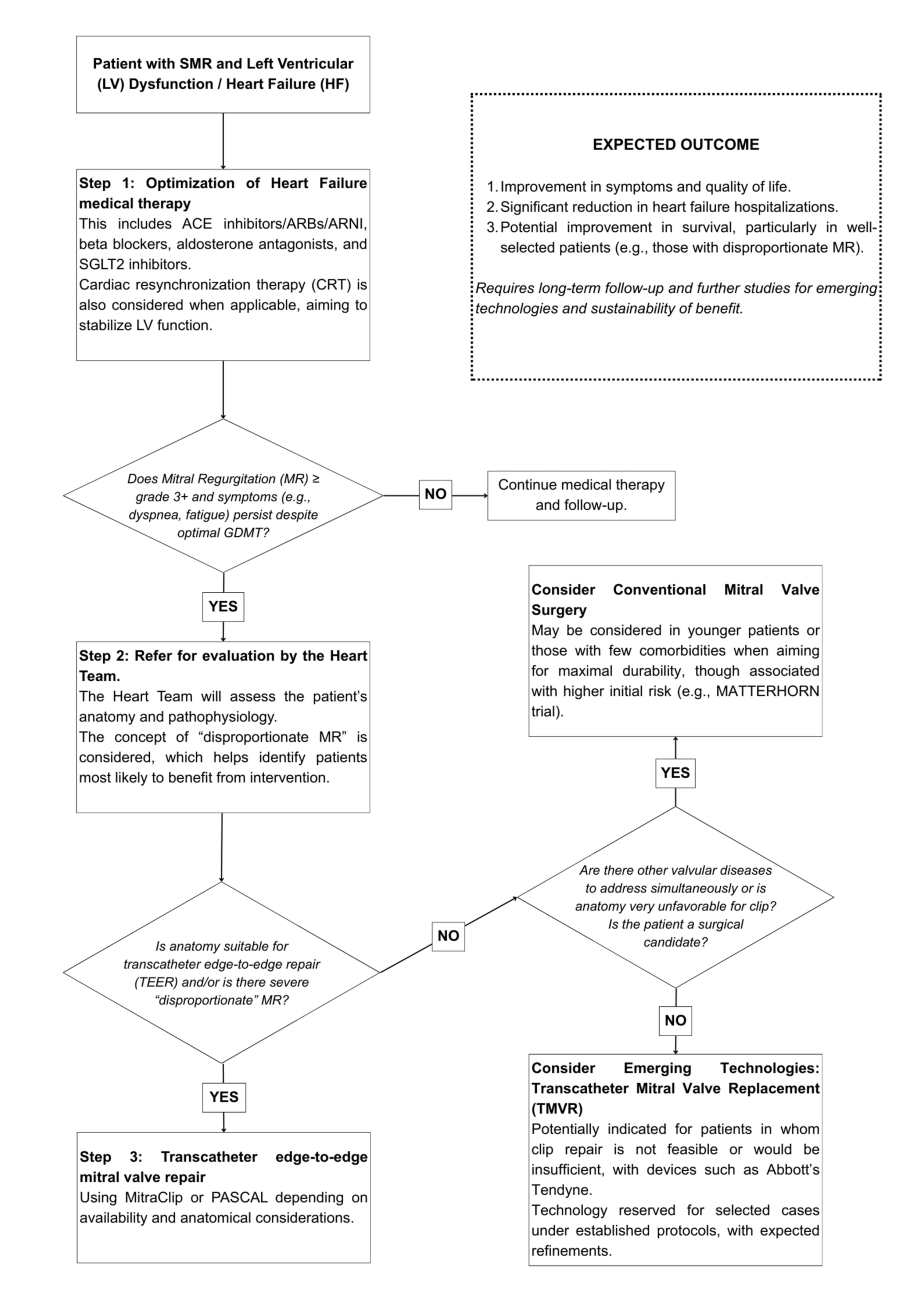
Proposed Clinical Decision-Making Framework for Secondary Mitral Regurgitation Stepwise overview of treatment selection in secondary mitral regurgitation, from guideline-directed medical therapy optimization to Heart Team evaluation and consideration of TEER, surgery, or TMVR in selected patients. MR, mitral regurgitation; GDMT, guideline-directed medical therapy; TEER, transcatheter edge-to-edge repair; TMVR, transcatheter mitral valve replacement.

Within this framework, decisions should be individualized within a multidisciplinary Heart Team, integrating anatomical suitability, the likelihood of durable MR reduction, the need for concomitant surgical treatment, surgical candidacy, and the overall clinical context. TEER remains an important option when anatomy is favorable and a less invasive strategy is preferred, whereas surgery may be advantageous in selected operable patients when a more complete or potentially more durable correction is expected. TMVR currently occupies a more limited and evolving role, mainly in patients who are not suitable for edge-to-edge repair or in whom clip-based treatment is expected to be insufficient. Figure 2 is intended as a schematic synthesis of these considerations rather than as a formal treatment guideline.

Strengths

This review provides an updated and clinically relevant synthesis of transcatheter therapies for SMR, incorporating pivotal randomized trials, contemporary meta-analyses, and selected complementary studies published through March 2026. By prioritizing high-level evidence while also incorporating targeted supporting studies on patient selection, reverse remodeling, and evolving transcatheter platforms, the manuscript offers a broad yet clinically focused overview of the current therapeutic landscape in SMR.

Another strength of this review is its integrative approach. Beyond summarizing efficacy and safety outcomes, the analysis contextualizes findings within key pathophysiological and clinical frameworks, including proportionate versus disproportionate regurgitation, optimization of guideline-directed medical therapy, and multidisciplinary decision-making. The inclusion of schematic figures and a practical decision-making framework further enhances the educational and translational value of the manuscript for clinicians involved in HF and structural heart disease care.

Limitations

This work has limitations inherent to its narrative design. Although the review prioritized randomized controlled trials and meta-analyses as the main sources of evidence, study selection was not conducted through a formal systematic review process, and predefined inclusion/exclusion criteria, duplicate screening, and structured risk-of-bias assessment were not applied. Therefore, some degree of selection bias cannot be excluded.

In addition, the review integrates evidence from studies with substantial heterogeneity in patient populations, definitions of SMR, imaging criteria, comparator groups, and follow-up duration. This is particularly relevant when comparing trials with discordant results or when interpreting pooled analyses that combine randomized and observational data. Furthermore, some sections addressing alternative TEER platforms, TMVR, and specific clinical subgroups rely on prospective cohorts, registry data, or post hoc analyses rather than dedicated randomized comparisons. These limitations should be considered when extrapolating the findings to broader clinical practice.

Future research directions

Despite current advances, several important questions remain unanswered. Further research is needed to clarify whether transcatheter correction of SMR translates into meaningful long-term survival benefit and sustained reduction in HF events. More precise identification of responders and nonresponders will also be essential, particularly through better integration of anatomical, echocardiographic, and clinical parameters such as LV size, myocardial strain, and overall HF burden.

Another relevant question is whether timely transcatheter correction of MR may, in selected patients, delay progression to advanced HF therapies such as LV assist devices or heart transplantation. In parallel, comparative studies will be needed to better define the relative role of TEER, surgery, and TMVR across different clinical and anatomical scenarios.

Other transcatheter strategies beyond TEER and TMVR also warrant further investigation. Among them, percutaneous mitral annuloplasty has shown proof-of-concept potential; for example, the sham-controlled REDUCE FMR (Carillon Mitral Contour System for Reducing Functional Mitral Regurgitation) trial demonstrated reductions in regurgitant volume and favorable ventricular remodeling in functional MR [50]. Future research should clarify how these and other next-generation approaches, including newer clip platforms, direct transcatheter annuloplasty, percutaneous neochordae, and TMVR, may expand treatment options and support more personalized therapeutic strategies tailored to each patient’s anatomy and pathophysiology.

## Conclusions

Transcatheter therapy has transformed the management of SMR in patients with HF. Current evidence supports MitraClip, in addition to optimal medical therapy, as an effective option to reduce HF-related hospitalizations in appropriately selected patients with severe SMR, while potential survival benefits appear less consistent across studies and may depend on patient selection and follow-up duration. Evidence also suggests that greater benefit may be achieved in individuals with “disproportionate” regurgitation relative to ventricular size, underscoring the importance of careful clinical and anatomical evaluation. Compared with surgery, transcatheter repair may offer comparable short-term outcomes with a more favorable early safety profile in selected patients, although treatment choice should remain individualized. Emerging technologies, including alternative TEER platforms and transcatheter mitral valve replacement, continue to expand the therapeutic landscape. Overall, current evidence supports TEER as a central component of SMR management in appropriately selected patients, while ongoing technological advances will likely further broaden transcatheter treatment options.
